# Curcumin Inhibits α-Synuclein Aggregation by Acting on Liquid–Liquid Phase Transition

**DOI:** 10.3390/foods13091287

**Published:** 2024-04-23

**Authors:** Jian-Feng Li, Zi-Qun Jiang, Sen Cao, Meng-Xin Zhang, Li-Hui Wang, Jun Liu, Yan-Hua Lu, Hong-Yan Wang, Xiao-Jing Hong, Zhi-Guo Wang, Jun-Ping Liu

**Affiliations:** 1 Institute of Ageing Research, School of Basic Medical Sciences, Hangzhou Normal University, 2318 Yuhangtang Road, Hangzhou 311121, China; lijianfeng@hznu.edu.cn (J.-F.L.); 2020111012028@stu.hznu.edu.cn (Z.-Q.J.); 2021111012043@stu.hznu.edu.cn (S.C.); 2022111026065@stu.hznu.edu.cn (M.-X.Z.); 20120001@hznu.edu.cn (L.-H.W.); 20180014@hznu.edu.cn (J.L.); 20120068@hznu.edu.cn (Y.-H.L.); 20193010@hznu.edu.cn (H.-Y.W.); 20197008@hznu.edu.cn (X.-J.H.); 2 Department of Immunology and Pathology, Faculty of Medicine, Central Clinical School, Monash University, Commercial Road, Prahran, VIC 3018, Australia

**Keywords:** Parkinson’s disease, α-synuclein, curcumin, anti-aggregation, molecular dynamics

## Abstract

Parkinson’s disease (PD), the second most common neurodegenerative disorder, is linked to α-synuclein (α-Syn) aggregation. Despite no specific drug being available for its treatment, curcumin, from the spice turmeric, shows promise. However, its application in PD is limited by a lack of understanding of its anti-amyloidogenic mechanisms. In this study, we first reconstructed the liquid–liquid phase separation (LLPS) of α-Syn in vitro under different conditions, which may be an initial step in entraining the pathogenic aggregation. Subsequently, we evaluated the effects of curcumin on the formation of droplets, oligomers, and aggregated fibers during the LLPS of α-synuclein, as well as its impact on the toxicity of aggregated α-synuclein to cultured cells. Importantly, we found that curcumin can inhibit amyloid formation by inhibiting the occurrence of LLPS and the subsequent formation of oligomers of α-Syn in the early stages of aggregation. Finally, the molecular dynamic simulations of interactions between α-Syn decamer fibrils and curcumin showed that van der Waal’s interactions make the largest contribution to the anti-aggregation effect of curcumin. These results may help to clarify the mechanism by which curcumin inhibits the formation of α-Syn aggregates during the development of PD.

## 1. Introduction

Parkinson’s disease (PD) is a common neurodegenerative disease. The aggregation of α-synuclein (α-Syn) is a main hallmark of PD [1,2,3], but specific pathological mechanisms remain unclear [4,5]. A number of studies have indicated that mutations in the α-Syn gene *SNCA* lead to familial PD, with the mutant α-Syn forming a major component of the Lewy bodies and Lewy neurites that characterize the brains of patients with PD [6,7]. α-Syn is a small protein of 140 amino acids, and its N-terminus (aa 1–60) is prone to forming an amphiphilic α-helix that has been shown to bind to Cu^2+^ ions [8] to mediate interactions of α-Syn with lipid membranes or proteins [9,10] and to facilitate α–Syn aggregation [11]. The non-amyloid β-component region (aa 61–95) has a strong tendency to form a β-sheet structure that is associated with the accumulation of α-Syn into fibril aggregates [12,13]. The flexible C-terminus (aa 96–140) is rich in acidic residues that mediate an irregular curl under physiologically relevant conditions, but deleting parts of the C-terminal region causes α-Syn aggregation in vitro [14]. However, the mechanisms mediating the aggregation of α-Syn remain unclear [15,16,17,18].

In particular, the early events of the aggregation process remain unclear. Ray et al. provided clues to these early steps by showing that α-Syn undergoes liquid–liquid phase separation (LLPS) prior to aggregation in vitro, and the resulting α-Syn liquid-like droplets eventually undergo a liquid-to-solid transition and form an amyloid hydrogel that contains oligomers and fibrillar species [2]. These findings suggest that phase separation acts as an initial trigger of α-Syn aggregation in PD pathology [19]. 

Curcumin, the main polyphenolic substance present in the rhizomes of *Curcuma longa* L. [20], has been shown to interact with amyloid-β peptide and α-Syn, thus inhibiting their aggregation, deposition, and neurotoxicity [21]. Importantly, curcumin has also been recognized for its pharmacological benefits in a multitude of pathological contexts, including diabetes, cancer, and even neurodegenerative diseases [22,23]. However, the use of curcumin as a treatment for PD has been limited by a lack of understanding of the mechanisms underlying its anti-amyloidogenic effects.

In this report, the process mediating α-Syn LLPS was investigated by analyzing the effects of molecular crowding, protein concentration, and pH on droplet formation as identified microscopically. In addition, we compared the LLPS process and its pH dependence of wild-type α-Syn to that of α-Syn bearing heritable mutations that confer an increased risk of PD. Then, we investigated the effect of curcumin both on droplet formation and of the aggregation of α-Syn during LLPS using multiple biochemical tools, as well as its impact on the toxicity of aggregated α-synuclein to cultured cells. Finally, molecular dynamic (MD) simulations were used to provide molecular insights into the interactions between curcumin and the pathogenic α-Syn decamer. We found that curcumin could inhibit the formation of α-Syn oligomers by affecting the initial phase transition of α-Syn in the condensation pathway through direct interaction with droplets. The findings here may help to clarify the mechanism by which curcumin inhibits the formation of α-Syn aggregates during the development of PD and provide insight into ways to improve treatment options.

## 2. Materials and Methods

### 2.1. Materials and Reagents

Polyethylene glycol 6000 (PEG-6000, catalog number: 528877), Thioflavin-T (ThT, catalog number: 596200), DMSO (catalog number: D8418), and curcumin (catalog number: C1386) were purchased from Sigma-Aldrich (St. Louis, MO, USA). Ni-NTA-agarose (catalog number: 30250) was purchased from Qiagen. Cy5.5-curcumin (catalog number: R-CUR-5) was purchased from Ruixibio (Xi’an, China). All other reagents were of analytical grade.

### 2.2. Expression and Purification of α-Syn Protein

Recombinant human α-Syn was expressed in *E. coli*. Briefly, the cDNA encoding either human α-Syn or GFP-tagged human α-Syn was cloned into a pET28a vector and transformed into *E. coli* BL21 (DE3).

The pellet from 200 mL culture was resuspended in 15 mL binding buffer (20 mM Tris, 500 mM NaCl, 20 mM imidazole, and 10 mM PMSF; pH 8.0) and lysed on ice by sonication at 400 W for 100 cycles (4 s working, 8 s free). The supernatant of the cell lysate resulting from centrifugation at 12,000× *g* at 4 °C for 20 min was applied to a Ni-NTA-agarose column. Wild-type (WT) proteins or the noted mutants containing 6× His tags were purified over Ni-NTA agarose. After extensive washing with binding buffer, the fusion protein was eluted with five-column volumes of elution buffer (20 mM Tris, 500 mM NaCl, and 50 mM imidazole, pH 8.0). The peak fractions containing the fusion protein were pooled and dialyzed overnight at 4 °C against 20 mM phosphate buffer (pH 7.4). The final solution was subsequently processed with pre-washed 10 kDa and 100 kDa cutoff filters (Merck Millipore, Darmstadt, Germany) to concentrate the protein and remove aggregates, respectively. Protein samples were analyzed using Coomassie staining following separation on 12% SDS-PAGE. Protein purity was analyzed using Bandscan software version 4.0 (BioMarin Pharmaceutical Inc., London, UK).

### 2.3. Liquid–Liquid Phase Separation Assay

Protein solutions were centrifuged at 12,000 rpm at 4 °C for 20 min to remove insoluble aggregates, and concentrations of soluble protein were measured by determining UV absorbance with a NanoDrop spectrophotometer (Thermo Fisher Scientific, Waltham, MA, USA). Reaction mixtures (100 μL), in which several factors were varied, including the concentration of protein (0, 10, 20, 40, or 80 μM), the concentration of PEG-6000 (0, 2.5, 5, 10, or 20%), and buffer pH (4.8, 5.8, 7.4, 8.0, or 8.8), were prepared. These reactions were prepared with wild-type α-Syn, with α-Syn bearing PD-related mutations (D2A, A30P, E46K, H50Q, and A53T) or with α-Syn bearing a phosphomimetic S129E mutation. The single amino acid substitutions for D2A, A30P, E46K, H50Q, A53T, and S129E were constructed by gene synthesis and confirmed by sequencing. To assay the effect of curcumin on the LLPS of α-Syn or its variants, reaction mixtures were prepared with various curcumin concentrations (0, 0.05, 0.10, 0.25, 0.50, 1.0, or 2.0 mM). GFP-tagged wild-type (WT) α-Syn or the noted PD-relevant mutants were induced to undergo LLPS in the presence of 10% PEG-6000 at the noted pH and in the presence of the noted concentrations of curcumin.

The solutions were deposited by drop-casting them onto modified glass slides and covering them with 12 mm coverslips. Images were taken with a DeltaVision Elite system on an Olympus IX71 inverted microscope running softWoRx 6.0. Differential interface contrast (DIC) and fluorescence images were acquired using a 60× objective lens with a CoolSnap HQ2 CCD camera. The mean numbers and diameters of droplets were determined from fluorescence images of three replicate samples using softWoRx 6.0.

### 2.4. DIC and Fluorescence Microscopy

The processes of phase separation and liquid droplet formation were visualized using a DeltaVision Elite system on an Olympus IX71 inverted microscope under DIC and fluorescence modes. For fluorescence-based studies, including analyses of FITC-labeled α-Syn or GFP-αSyn fusion proteins, liquid droplets were observed using appropriate fluorescence channels (488 nm for FITC, GFP, and Thioflavin-T (*ThT*); 555 nm for LysoTracker dye). In each case, control experiments were measured in parallel to determine baseline fluorescence settings [24]. Images of size 1024 × 1024 pixels were acquired with a DeltaVision^®^ fluorescent microscope with 60× objective at room temperature. Images were further processed via de-convolution and quick projection methods to improve the quality using softWoRx 6.0 software “https://download.cytivalifesciences.com/cellanalysis/download_data/softWoRx/softworx_downloads.htm (accessed on 25 January 2024)”.

### 2.5. Co-Localization of Curcumin and α-Syn during Droplets Formation

The co-localization of curcumin and α-Syn during droplet formation was visualized using the EVOS Automated Cell Imaging System (ThermoFisher Scientific, Waltham, MA, USA). Briefly, GFP-tagged α-Syn (80 μM) was induced to undergo LLPS in the presence of 10% PEG-6000 and in the presence of 10 μM Cy5.5-curcumin. For fluorescence-based studies, including the analyses of Cy5.5-labeled curcumin or GFP-αSyn fusion proteins, liquid droplets were observed using the appropriate fluorescence channels (470/525 nm for GFP and 628/692 nm for Cy5.5).

### 2.6. Thioflavin-T Fluorescence Assay

To detect amyloid fibril growth, an assay based on ThT fluorescence was performed. Solutions containing α-Syn (40 μM) with or without 10% PEG-6000 or curcumin were incubated for designated time periods. Aliquots (25 μL) of these samples were added to 25 μL of a 200 μM ThT solution. This mixture was further diluted to 100 μL using a PBS buffer (pH 7.4). The resulting fluorescence was determined using a Multiskan FC Microplate Reader (Thermo Scientific, Waltham, MA, USA) with excitation and emission wavelengths set at 450 nm and 480 nm, respectively. Each experiment was repeated at least three times. Thioflavin T (ThT)-based assays were performed in the presence of increasing concentrations of curcumin in order to monitor the kinetics of the aggregation of wild-type α-Syn. Control samples lacked PEG-6000 and curcumin. All other samples included 10% PEG-6000.

### 2.7. Native PAGE

Protein samples were prepared under non-denaturing conditions and separated by non-denaturing PAGE at 120 V [25]. The proteins were then transferred electrophoretically to polyvinylidene fluoride membranes. The membranes were blocked in TBS-T containing 5% non-fat milk and then incubated with anti-α-Syn antibodies (catalog number: SAB4502829, Sigma-Aldrich). The negative control lacked curcumin or PEG-6000.

### 2.8. Cell Viability Assay

The colorimetric assay was used to detect the effects of curcumin on the viability of cultured cells treated with aggregated α-Syn. The aggregated protein was prepared according to the method described previously [26]. Briefly, α-Syn (5 mg/mL) was incubated in 10% PEG with or without 50 μM curcumin for 3 days at 37 °C with agitation at 1000 rpm. HEK 293 cells were seeded at a density of 5 × 10^4^ cells per well in 96-well plates. After incubation at 37 °C and 5% CO_2_ for 24 h, the cells were treated with the solutions of aggregated α-Syn and incubated for another 24 h. MTT (20 μL at 5 mg/mL) was then added to each well, and the plates were incubated at 37 °C for 4 to 8 h. After the removal of the supernatant, dimethyl sulfoxide (200 μL) was added to dissolve the formazan product that remained in the wells. After 30 min, the absorbance was measured with a Multiskan FC Microplate Reader (Thermo Scientific) at a wavelength of 570 nm. The results were recorded by averaging at least three repeated experiments, and the absorbance of control cells was taken as 100% cell viability.

Fluorescence microscopy was used to trace the fate of labeled α-Syn in living cells following oligomerization under various conditions. GFP-αSyn was pre-incubated for 3 d without PEG-6000 or curcumin (Ctrl α-Syn), with 10% PEG-6000 (LLPS α-Syn), or with 10% PEG-6000 and 50 μM curcumin (LLPS α-Syn + Cur) and then applied to HEK293 cells for 0.5 h. Immediately after this incubation or 24 h later, the cells were counterstained with LysoTracker and DAPI to visualize lysosomes and nuclei, respectively.

### 2.9. Molecular Simulations

Molecular dynamic simulations were performed to investigate the interactions between α-Syn and curcumin. The coordinates of the structure of an α-Syn decamer fibril were retrieved from the protein data bank (2N0A) [27]. The central parallel β-sheet region encompassing residues 38 to 98 (α-Syn^38–98^) was isolated, and a structure containing ten of these regions was set as the receptor (denoted as 10-mer). The structure of the curcumin molecule was generated with GaussView software (version 6.0.16) [28] and optimized with Gaussian 16 software [29] using the density functional theory at the level of 6–31G(d).

Five or ten curcumin molecules were randomly positioned within 8.0 Å of 10-mer α-Syn^38–98^ with Packmol software version 20.14.3 [30]. The structures of apo 10-mer and those with five or ten curcumin molecules bound (10-mer + 5 Cur and 10-mer + 10 Cur) were individually immersed into the centers of truncated octahedron boxes of TIP3P water molecules with margin distances of 10.0 Å. MD simulations were conducted with AMBER 22 software [31]. The FF14SB force field was applied to α-Syn^38–98^. For curcumin, the RESP charge, calculated according to a reported procedure [32], was used in combination with the AMBER GAFF2 [33] force field parameter. Chloride counterions were added to maintain charge neutrality. A 400 ns production of MD simulations and subsequent molecular mechanics/generalized Born surface area (MM/GBSA) calculations for binding affinity evaluation were performed by following our previous reports [34].

### 2.10. Statistical Analysis

All experiments were performed in triplicate. Quantitative values were expressed as mean ± SE. Statistical analyses were performed using Student’s *t*-test (STATISTICA version 13.3, Statsoft Inc., Tulsa, OK, USA) on a conventional personal computer. One-way ANOVA was applied to determine significant differences, with the level of significance defined as *p* < 0.05.

## 3. Results and Discussion

### 3.1. Purification of α-Syn Protein

**Figure 1:**
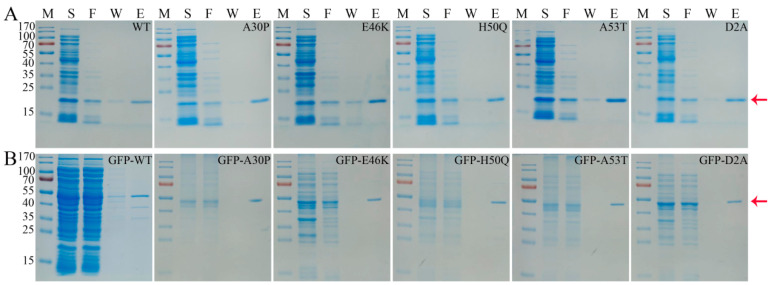
Purification of recombinant human α-Syn and GFP-αSyn proteins. α-Syn (**A**) or GFP-αSyn (**B**) wild-type (WT) proteins or the noted mutants containing 6× His tags were purified over Ni-NTA agarose. Protein samples were analyzed with Coomassie staining following separation on 12% SDS-PAGE. The expected sizes of α-Syn and GFP-αSyn were 17 kDa and 45 kDa, respectively. Protein purity was analyzed using Bandscan software (BioMarin Pharmaceutical Inc., London, UK). Lane M: molecular weight marker (kDa); lane S: supernatant of cell lysate; Lane F: Ni-NTA agarose flow-through; Lane W: final column wash; Lane E: elution. Arrow indicates the purified protein.

Recombinant human α-Syn and α-Syn with an N-terminal GFP tag were purified following expression in bacteria as 6× His fusion proteins. As shown in Figure 1 and Figure S1, most of the non-specific bacterial proteins were removed upon extensive washing with a buffer containing 20 mM imidazole, and the target proteins were completely eluted with a buffer containing 50 mM imidazole. In each case, the final purity of the purified protein was determined to be greater than 90% using SDS-PAGE analyses (Figure 1 and Figure S1).

### 3.2. Investigation of Liquid–Liquid Phase Separation in the Aggregation of α-Syn

An important step in the formation of α-Syn aggregates may be the separation of the protein into liquid-phase droplets. In order to study this process in vitro, we performed microscopic analyses in which fluorescently labeled recombinant human α-Syn was incubated under various conditions, and then fluorescence images to DIC images of the resulting droplets were compared. First, we prepared the proteins using affinity purification and size exclusion filtration and determined the effect of the fluorescent labeling of α-Syn on the LLPS process. The droplets formed by FITC- or GFP-labeled α-Syn were similar in number or morphology as compared to those formed by unlabeled α-Syn (Figure S2). In addition, the droplets formed by both fluorescently labeled α-Syn were highly mobile and underwent frequent fusion events with a rapid relaxation into spherical assemblies (Figure S3). These results suggest that LLPS in vitro using fluorescently labeled α-Syn reflects the characteristics of LLPS formation by native α-Syn.

**Figure 2:**
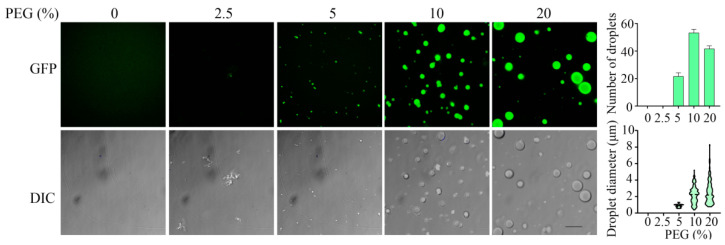
In vitro microscopic analysis of LLPS of α-Syn. Representative fluorescence (**top**) and DIC (**bottom**) images of droplets of GFP-αSyn that underwent phase separation in the presence of the noted concentrations of PEG-6000 at pH 7.4 are shown in the left-hand series of panels (Scale bar, 10 μm). The right-hand panel displays the mean numbers (**top**) and diameters (**bottom**) of droplets as determined from fluorescence images of 3 replicate samples using softWoRx 6.0.

Auto-inhibitory intramolecular interactions and long-range intermolecular interactions are key factors that have been shown to influence phase transitions in other systems [35]. Therefore, we predicted that increasing molecular crowding via the addition of PEG-6000 would increase intermolecular interactions of α-Syn and promote phase transition. To investigate the effect of molecular crowding on the α-Syn LLPS, then, droplet formation was assayed in the presence of increasing concentrations of PEG-6000. As shown in Figure 2, while little droplet formation was observed when 40 μM α-Syn was incubated in the absence of PEG-6000 or in the presence of 2.5% PEG-6000, obvious droplet formation did occur when 5% PEG-6000 was included in the mixture. Increasing the concentration of PEG-6000 to 10% or 20% led to the formation of more droplets, and the mean diameter of the droplets increased with increasing concentrations of PEG-6000. As molecular crowding was found to enhance droplet formation [2], we investigated the effect of protein concentration on LLPS. As shown in Figure S4, at a physiological pH level, increasing the concentration of α-Syn in the presence of 10% PEG-6000 increased droplet formation, especially in terms of mean droplet diameter.

**Figure 3:**
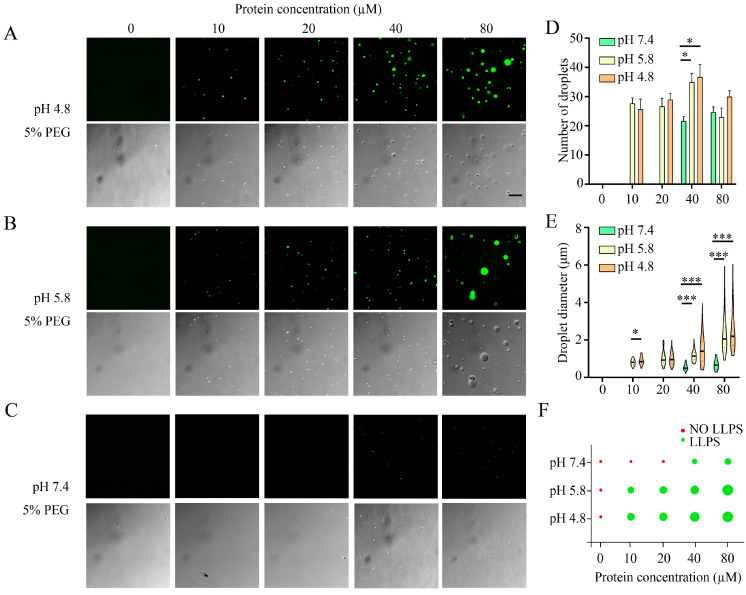
Effects of protein concentration and pH on the LLPS behavior of α-Syn. Representative fluorescence and differential interference contrast (DIC) images of droplets produced by the phase separation of GFP-αSyn incubated at the noted protein concentrations at pH 4.8 (**A**), pH 5.8 (**B**), or pH 7.4 (**C**) (Scale bar, 10 μm). The right-hand panel displays the mean numbers (**D**) and diameters (**E**) of droplets as determined from the fluorescence images of 3 replicate samples using softWoRx 6.0. Regime diagram illustrating the phase separation of GFP-αSyn at different protein concentrations at pH 4.8, pH 5.8, or pH 7.4 (**F**). * means *p* < 0.05, *** means *p* < 0.001.

**Figure 4:**
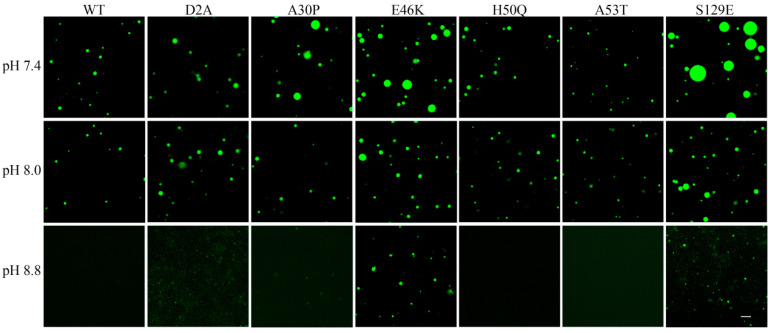
Effects of mutations and pH on the liquid–liquid phase separation behavior of α-Syn. Representative fluorescence microscopic images of droplets of wild-type (WT) or mutant GFP-αSyn that underwent phase separation in the presence of 10% PEG-6000 at the indicated pH (Scale bar, 50 μm).

At the same time, we also investigated the dependence of the LLPS on pH. The isoelectric point (pI) of native α-Syn has been shown to be approximately 4.7 (pI 5.8 of GFP-αSyn) [2], so it is possible that stronger droplet formation would occur near acidic pH. As shown in Figure 3A–E, the acidic environment was favorable for droplet formation, and the critical concentration of α-Syn LLPS in the presence of 5% PEG-6000 was reduced from approximately 40 μM at pH 7.4 (Figure 3C,F) to approximately 10 μM at pH 5.8 (Figure 3B,F). Conversely, we found that increasing the pH to 8.8 completely inhibited the phase transition of wild-type α-Syn (Figure 4). Thus, in agreement with a previous report [2], adjusting the pH of the solution to a value close to the pI of the protein reduced the critical concentration of α-Syn LLPS, as non-specific intermolecular interactions were favored when the overall charge of the protein was near neutral.

### 3.3. Effects of Parkinson’s Disease-Relevant Mutations on Droplet Formation

Heritable mutations and Ser129 phosphorylation are known to play major roles in α-Syn LLPS and aggregation in PD pathogenesis [2]. Therefore, we studied the effects of PD-related mutants (D2A, A30P, E46K, H50Q, and A53T) and the phosphomimetic mutation S129E on α-Syn LLPS in vitro under various pH conditions. Three mutations (A30P, E46K, and S129E) were found to promote α-Syn LLPS at physiological pH, as shown by the increased numbers or diameters of droplets formed at pH 7.4 (Figure 4 and Figure S5). In addition, in comparison to wild-type α-Syn, the introduction of the E46K and S129E mutations led to α-Syn proteins that underwent the phase transition at pH 8.8 (Figure 4). These findings suggest that at least two of the common PD-related heritable mutations, as well as Ser129 phosphorylation, may accelerate amyloid aggregation by increasing the pH-influenced phase transition of α-Syn.

### 3.4. Effect of Curcumin on Droplet Formation

**Figure 5:**
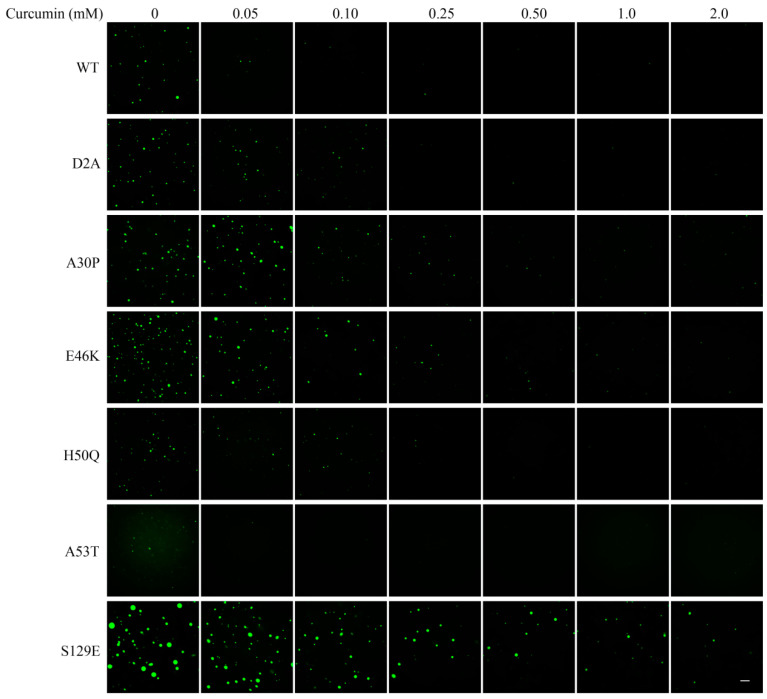
Effect of curcumin on the LLPS behavior of wild-type and mutant α-Syn. GFP-tagged wild-type (WT) α-Syn or the noted PD-relevant mutants were induced to undergo LLPS in the presence of 10% PEG-6000 at the noted pH and in the presence of the noted concentrations of curcumin. Samples with 1 or 2 mM curcumin included 20% DMSO to improve solubility; this concentration of DMSO was found to have no effect on phase transition behavior (Scale bar, 25 μm).

Because curcumin has been shown to inhibit the aggregation of α-Syn [36], we investigated the impact of this compound on the α-Syn phase transition. Consistent with a previous report [36], curcumin at low concentrations (<25 μM) had no observable effect on the number or morphology of α-Syn droplets. However, as shown in Figure 5, the phase transition of wild-type α-Syn at pH 8.0 was inhibited when the concentration of curcumin was increased to 50 μM. In addition, we also found that the inclusion of at least 50 μM curcumin also inhibited the LLPS of three PD-related mutants (D2A, H50Q, and A53T), while higher doses of curcumin were needed to affect the inhibition of the LLSP of α-Syn with either of two other heritable PD-related mutations (A30P or E46K) or with a mutation mimicking Ser phosphorylation (S129E) (Figure 5). Droplet formation of the Ser-phosphorylation mimic was even observed at concentrations of curcumin up to 2 mM (Figure 5). It should be noted that 20% DMSO was included in experiments involving higher concentrations of curcumin (1 mM and 2 mM) to improve solubility; however, we determined that 20% DMSO had no effect on the phase transition.

Notably, the curcumin-resistant mutations (A30P, E46K, and S129E) were also associated with more intense phase transitions relative to the wild-type protein (Figure 4). This correlation suggests that the differences in the LLPS-inhibitory effects of curcumin on wild-type or mutant α-Syn are related to the intensity of the phase transition experienced in the absence of curcumin.

### 3.5. Co-Localization of Curcumin and α-Syn during Droplets Formation

In order to determine if curcumin could bind directly to the liquid droplets of α-Syn, we investigated the co-localization of curcumin and α-Syn during droplet formation. As shown in Figure S6, compared with GFP control, α-Syn could form liquid droplets in the presence of 10% PEG-6000, and Cy5.5-labeled curcumin could co-localize with liquid droplets efficiently. Unlike previous studies of the interaction of curcumin and α-Syn by fluorescence or nuclear magnetic resonance (NMR) spectroscopy [37], our experiment provides an alternative way to study the interaction of them. However, the disadvantage of this approach was that the conformation of α-Syn in the condensates cannot be controlled.

### 3.6. Effect of Curcumin on the Aggregation of α-Syn during LLPS

**Figure 6:**
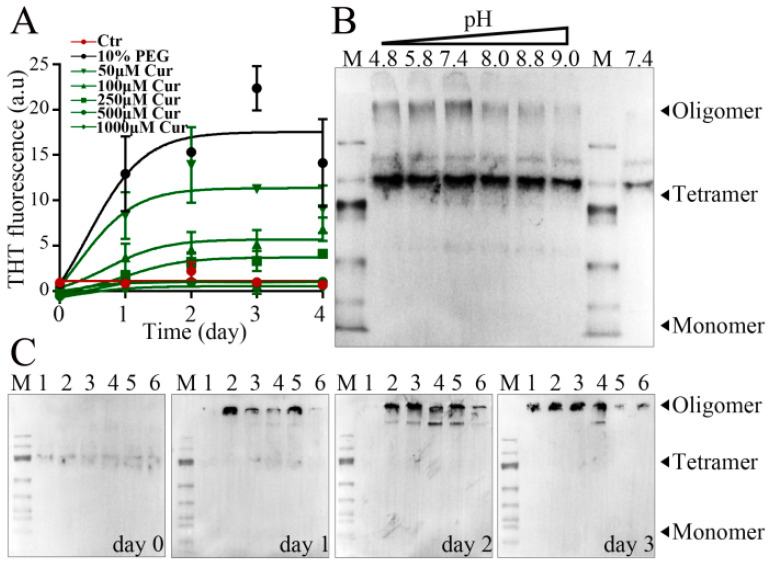
Effects of curcumin and pH on the aggregation of α-Syn in the liquid–liquid phase separation process. (**A**) Thioflavin T (ThT)-based assays were performed in the presence of increasing concentrations of curcumin in order to monitor the kinetics of the aggregation of wild-type α-Syn. A higher fluorescent signal indicates a greater extent of aggregation. Control samples (red line) lacked PEG-6000 and curcumin. All other samples included 10% PEG-6000. Data are presented as mean ± SE (n = 3). (**B**) Oligomerization in the absence (Lane 9, the rightmost lane) or presence (Lanes 2–7, lanes with pH values above) of 10% PEG-6000 at the noted pH was monitored by native PAGE, followed by immunoblotting with an anti-α-Syn antibody. The migration distances of monomeric, tetrameric, and oligomeric α-Syn are indicated with arrows. (**C**) Native PAGE and immunoblotting analyses of the time-dependent effects of curcumin on oligomer formation. Lane 2 (lane with number of 1 above): negative control without curcumin or PEG-6000; Lanes 3–7 (lanes with numbers of 2–6 above): 0, 50, 100, 250, or 500 μM curcumin, respectively, with 10% PEG.

In order to provide additional insight into the mechanism of the inhibition of the aggregation of α-Syn by curcumin, we further probed this effect using the ThT fluorescence assay and native gel electrophoresis. In the ThT fluorescence assay, which is commonly used to study fibril formation in vitro, the fluorophore has a higher affinity for fibrils than for monomeric proteins, resulting in increased fluorescence upon the formation of aggregates. As shown in Figure 6, this assay confirmed that the LLPS induced by 10% PEG-6000 promotes the aggregation of α-Syn into fibrils; in addition, we found that the inclusion of curcumin led to a dose-dependent decrease in the PEG-6000-induced fluorescence increase (Figure 6A), suggesting that the anti-LLPS effects of curcumin also result in decreased fibril formation.

The effect of phase transition on oligomer formation was examined using native PAGE, followed by immunoblotting for α-Syn. As shown in Figure 6B, this assay revealed the formation of oligomers following incubation under conditions that support the LLPS process. For example, the formation of oligomers was observed to be stronger in the presence of 10% PEG-6000 than in its absence (Figure 6B). In addition, as observed in microscopic assays of the LLPS process (Figure 4), the formation of the oligomers of the wild-type protein can be observed at physiological 7.4, with a particularly strong inhibition of oligomer formation at higher pH values (Figure 6B). Because curcumin was found to inhibit LLPS of α-Syn, we further examined the effect of curcumin on the formation of α-Syn oligomers. As shown in Figure 6C, compared with control experiments containing 10% PEG-6000 but lacking curcumin, curcumin clearly inhibited the formation of oligomers in a dose-dependent manner when analyzed after 24 h incubation with 10% PEG-6000. Although oligomers did form as the samples were incubated for additional time (days 2 and 3), this process was inhibited by curcumin (Figure 6C and Figure S7). Considering the inhibition of both LLPS and oligomerization by curcumin, we conjecture that curcumin inhibits amyloid formation by inhibiting the occurrence of LLPS and the subsequent formation of the oligomers of α-Syn in the early stages of aggregation.

In contrast to the traditional deposition pathway, the condensation pathway is another route to form amyloid aggregates, in which α-Syn undergoes LLPS, promoting the amyloid aggregation in the condensates [2,3]. Nevertheless, how curcumin modulates α-Syn amyloid aggregation in the condensation pathway remains unexplored. Furthermore, the relationship between curcumin and α-Syn fibrillization remains unclear due to the different effects observed under different experimental conditions [38]. Some research indicates curcumin suppresses α-Syn fibrosis [39,40,41,42], whereas other studies suggest it can expedite α-Syn fibrosis, leading to morphologically distinct amyloid fibers [37,38]. Here, we investigated the impact of curcumin on α-Syn amyloid aggregation under LLPS conditions. Our findings revealed that low concentrations of curcumin exerted no significant effect on the number or morphology of α-Syn droplets, aligning with prior studies [36]. However, curcumin in high concentrations exhibits a remarkable inhibitory effect on the formation of α-Syn droplets under LLPS conditions. This is important, suggesting that curcumin may play a role in the initial phase transition of α-Syn in the condensation pathway, which will provide new insights into ways to improve treatment options.

### 3.7. Effect of Curcumin on the Cytotoxicity of α-Syn Aggregates

In order to determine if the inhibitory effect of curcumin on α-Syn aggregation is sufficient to protect cells against α-Syn-mediated cytotoxicity, we investigated the interactions of α-Syn with HEK293 cells. First, we used MTT assays to quantify the effects of α-Syn on cell viability. Here, we found that the incubation of HEK293 cells with α-Syn that had been oligomerized in the presence of 10% PEG-6000 led to a drastic decrease in cellular viability. However, the inclusion of 50 μM curcumin into the oligomerization samples led to a 20% increase in the viability of cells exposed to the α-Syn solution, suggesting that the curcumin-mediated inhibition of oligomerization decreased the toxicity of the α-Syn sample.

**Figure 7:**
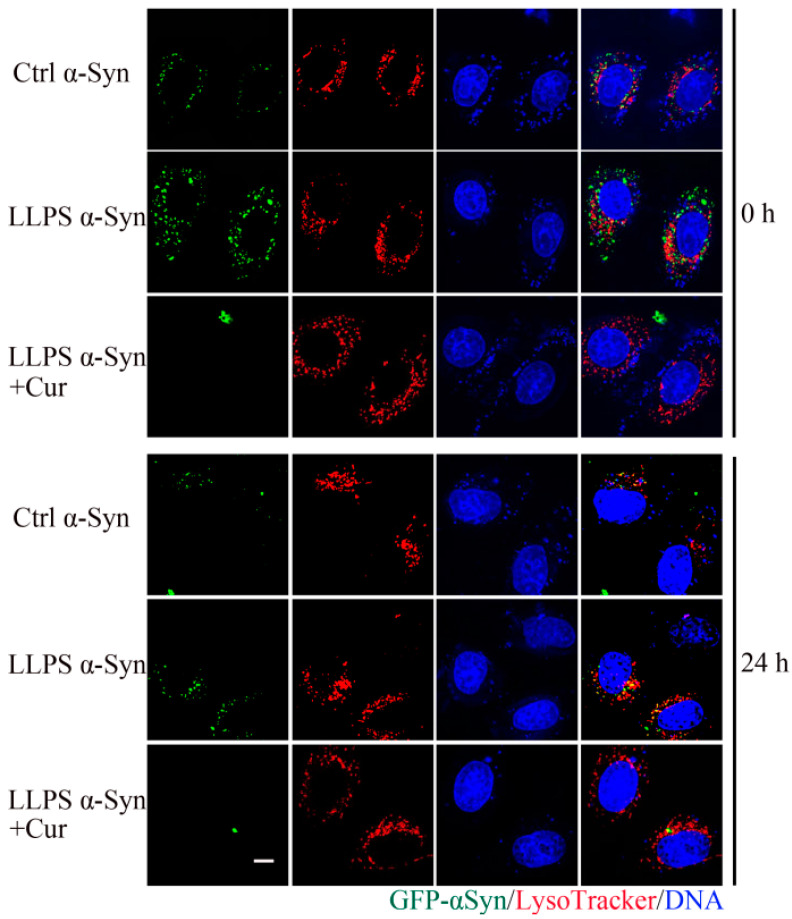
Effect of curcumin on the entry of exogenous α-Syn into living cells. GFP-αSyn was pre-incubated for 3 d without PEG-6000 or curcumin (Ctrl α-Syn), with 10% PEG-6000 (LLPS α-Syn) or with 10% PEG-6000 and 50 μM curcumin (LLPS α-Syn + Cur) and then applied to HEK293 cells for 0.5 h. Immediately after this incubation or 24 h later, the cells were counterstained with LysoTracker and DAPI to visualize lysosomes and nuclei, respectively, and the cells were observed with a fluorescence microscope.

Oligomerization has been shown to speed the internalization of α-Syn into cells and to slow its degradation in lysosomes. Therefore, we used fluorescence microscopy to trace the fate of labeled α-Syn in living cells following oligomerization under various conditions. Here, we first incubated GFP-αSyn for three days in the presence of 10% PEG-6000 and various concentrations of curcumin and then incubated HEK293 cells with these samples for 0.5 h, followed by extensive washing of the cells. The cells were observed using fluorescence microscopy immediately after washing and then 24 h later. As shown in Figure 7, compared with α-Syn incubated in the absence of PEG-6000, more of the phase-changed α-Syn protein was found to enter the cells, and it was found to degrade more slowly in lysosomes once internalized. In addition, the co-incubation of α-Syn with both PEG-6000 and curcumin was found to reduce the amount of α-Syn protein entering the cells relative to aggregated protein incubated in the absence of curcumin. These results confirmed the relevance of the inhibition of oligomerization by curcumin; however, while we have shown that curcumin affects the initial phase transition, we are unable to exclude the possibility that curcumin also affects other aspects of α-Syn oligomerization, for example, through the induction of conformational changes that could slow fibril formation.

### 3.8. Insights into Molecular Interactions Derived from Molecular Simulations

**Figure 8:**
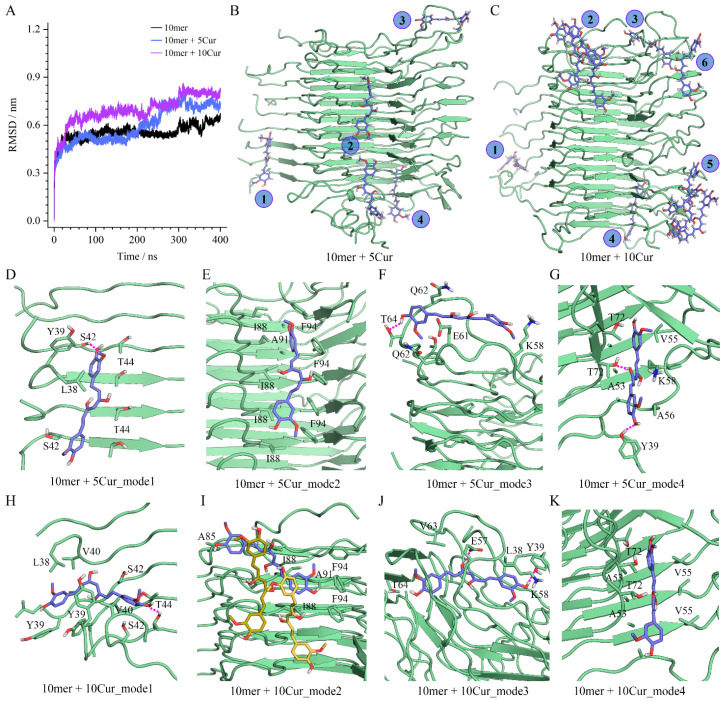
Molecular dynamic simulations of an oligomer of α-Syn fragment and the complexes of this oligomer with curcumin molecules. (**A**) The root mean squared deviation (RMSD) profiles across the 400 ns MD simulations. (**B**,**C**) The overall MD-equilibrated binding conformations of a 10-mer of α-Syn^38−98^ with 5 (**B**) or 10 (**C**) curcumin molecules. The curcumin molecules are shown as blue sticks, and the binding sites are indicated using Arabic numerals. (**D**–**K**) The binding modes of the curcumin molecules in the noted models, with the curcumins and nearby amino acids shown in blue and green sticks, respectively. Intermolecular hydrogen bonds are indicated with magenta dotted lines. In panel (**I**), adjacent curcumin molecules that form stacking interactions with the directly bound curcumins are shown as yellow sticks.

The interactions between α-Syn^38–98^ decamer and curcumin molecules were investigated at the atomic level using MD simulations. As shown in Figure 8, relative to the apo form of the protein oligomers, the binding of curcumin was found to elevate the RMSD values of 10-mer α-Syn^38–98^ in a dose-dependent manner (Figure 8A), consistent with a dynamic destabilization of the oligomers by curcumin. The RMSF profiles uncovered that the terminal residues showed the most significant conformational fluctuations. Moreover, relative to the 5 Cur-bound 10-mer α-Syn^38–98^, the 10 Cur-bound 10-mer α-Syn^38–98^ was of more flexibility, especially for the residues located in the outmost monomers (Figure S8). Compared with the initial NMR structure, the curcumin-bound α-Syn^38–98^ showed increased solvent-accessible surface area (Figure S9), indicative of conformational alternations. Though the 5 Cur- and 10 Cur-bound 10-mer α-Syn^38–98^ showed similar mass-weighted radius of gyration (Rg), at the final equilibrated stage, the max Rg of the 10 Cur-bound 10-mer α-Syn^38–98^ (4.47 nm) is larger than that of the 10 Cur-bound 10-mer α-Syn^38–98^ (4.30 nm), indicating a more dramatic conformational change (Figure S10). From the MD-equilibrated conformations of complexes involving 10-mer α-Syn^38–98^ with either 5 (10-mer + 5 Cur; Figure 8B) or 10 (10-mer + 10 Cur; Figure 8C)-bound curcumin molecules, four shared binding modes were identified in both complexes. The identification of two additional binding modes in the 10-mer + 10 Cur binding complex was consistent with the increased anti-aggregation effect of increasing concentrations of curcumin (Figure 8B,C). A more detailed inspection of the binding conformations (Figure 8D–K) showed variability in the orientation of the curcumin molecules even in places where the same binding mode and the same binding residues are observed. In these cases, the orientation of the curcumin molecule was also shown to impact its binding affinity (Table S1). For instance, the binding affinity of mode2 in 10-mer + 10 Cur (−42.71 kcal/mol) was slightly weaker than that of 10-mer + 5 Cur (−44.54 kcal/mol) (Figure 8E,I), and the curcumin molecules of mode3 (Figure 8F,J) showed a wider divergence in binding affinity, which were −49.66 and −32.35 kcal/mol in 10-mer + 10 Cur and 10-mer + 5 Cur, respectively (Table S1).

In terms of specific non-covalent interactions, MM/GBSA calculations revealed that van der Walls interactions made the largest contributions to the binding of curcumin to the decamer in all cases, indicating the pivotal role of the phenyl groups of curcumin. We also identified important intermolecular hydrogen bonds between the phenolic hydroxyl groups of curcumin and α-Syn^38–98^ residues Y39, S42, T44, E57, T64, and T72 (Figure 8D,F–H,J). The hydrogen bond analysis in Figure S11 demonstrated that at the final equilibrated stage of MD simulations, the number of intermolecular hydrogen bonds in 10-mer + 10 Cur (11.61) is much more than that in 10-mer + 5 Cur (6.89), agreeing with the overall enhanced binding affinity and anti-aggregation effect of curcumin under a higher concentration. In addition, the Ramachandran plots displayed that the proportions of the residues in most favored regions were 89.4%, 83.8%, and 80.8% for the NMR 10-mer structure (2N0A), the 5 Cur-bound 10-mer, and the 10 Cur-bound 10-mer, respectively (Figure S12), further supporting the dose-dependent effect of curcumin on the aggregated α-Syn.

## 4. Conclusions

According to results from our in vitro phase transition assay, as well as other biochemical assays of α-Syn oligomerization, we conclude that a primary effect of curcumin on α-Syn oligomerization involves the inhibition of the initiation of the LLPS process. In addition, MD simulations allowed us to conclude that van der Waals interactions between curcumin and the α-Syn decamer provide the largest contribution to this anti-aggregation effect of curcumin. These results show that curcumin inhibits amyloid formation in part by inhibiting the occurrence of LLPS and the subsequent formation of the oligomers of α-Syn in the early stages of aggregation. Clarifying the mechanism by which curcumin inhibits the formation of α-Syn aggregates early in the aggregation process provides insight into ways to improve treatment options. Further work is required to determine the effect of curcumin on the oligomerization of the clinically relevant mutations of α-Syn.

## Data Availability

The original contributions presented in the study are included in the article/Supplementary Materials, further inquiries can be directed to the corresponding authors.

## Supplementary Materials

The supporting information can be downloaded at https://www.mdpi.com/article/10.3390/foods13091287/s1.
