# Supplementary material for: Curcumin Inhibits α-Synuclein Aggregation by Acting on Liquid–Liquid Phase Transition
Source: Foods. 2024 Apr 23;13(9):1287. doi: 10.3390/foods13091287 (PMC11083653; doi:10.3390/foods13091287)
Supplement: Supplementary file 1 [file foods-13-01287-s001.zip › foods-2938642-supplementary.pdf]

Supporting Information

Table S1. Binding free energies between 10mer α-Syn$^{38\text{-}98}$ and curcumins under the shared four kinds of binding modes evaluated with MM/PBSA approach.[a]

Complex	Mode	Energy components				
		ΔE_{ele}	ΔE_{vdW}	ΔG_{GB}	ΔG_{SA}	ΔG_{bind}
10mer + 5Cur	mode1	-6.71 ± 1.98	-44.64 ± 3.26	9.96 ± 1.75	-5.79 ± 0.28	-47.18 ± 3.34
	mode2	-4.21 ± 1.45	-42.13 ± 2.52	8.05 ± 1.25	-6.26 ± 0.17	-44.54 ± 2.51
	mode3	-3.14 ± 1.86	-30.42 ± 6.07	6.03 ± 1.78	-4.83 ± 0.84	-32.35 ± 6.43
	mode4	-5.21 ± 1.16	-48.29 ± 3.67	8.18± 0.99	-6.60 ± 0.22	-51.93 ± 3.77
10mer + 10Cur	mode1	-4.09 ± 2.11	-37.47 ± 6.56	7.48 ± 2.17	-5.56 ± 0.99	-39.65 ± 6.90
	mode2	-2.31 ± 0.86	-40.26 ± 3.16	6.04 ± 0.81	-6.17 ± 0.28	-42.71 ± 3.05
	mode3	-6.31 ± 1.82	-46.66 ± 3.81	9.60 ± 1.54	-6.30 ± 0.56	-49.66 ± 4.12
	mode4	-0.43 ± 0.64	-12.16 ± 1.39	1.68 ± 0.54	-1.94 ± 0.17	-12.85 ± 1.46

[a] Energies are in kcal/mol.

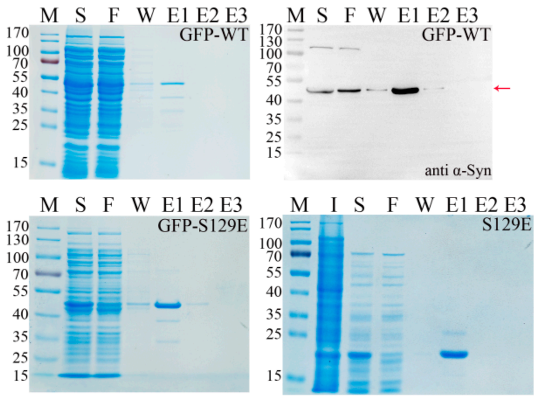

Fig. S1. Western blot analysis of GFP/α-Syn and purification of α-Syn mutant S129E.

The elution efficiency of elution buffer containing different imidazole concentration was detected by 12% SDS-PAGE, and the target protein of GFP-WT (same image in Fig 1) or mutant S129E can be completely eluted with 50 mM imidazole. Lane M: molecular weight marker; lane S: supernatant of cell lysate; lane I: induced whole cell; lane F: Flow-through; lane W: Wash; lane E1: Elution with 50 mM imidazole; lane E2: Elution with 100 mM imidazole; lane E3: Elution with 250 mM imidazole.

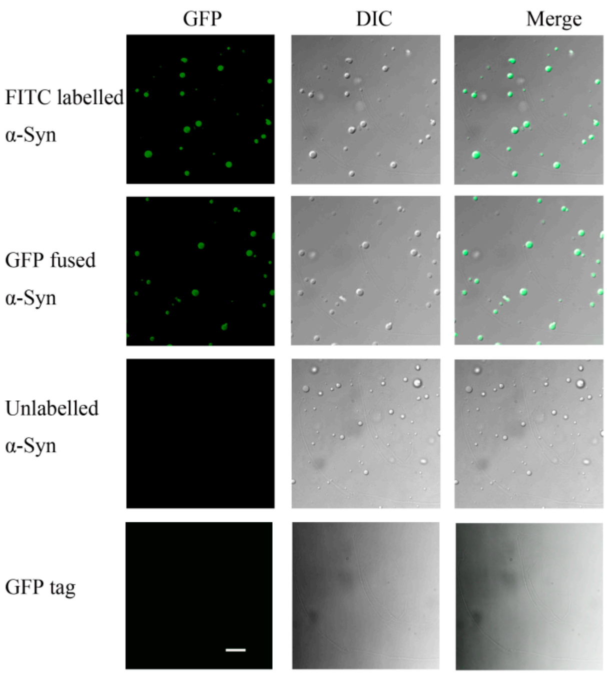

Fig. S2. The effect of fluorescence labeling on α-Syn LLPS.

The droplets formation of FITC labelled or GFP-fused α-Syn is same as that of unlabeled α-Syn, scale bar 10 μm.

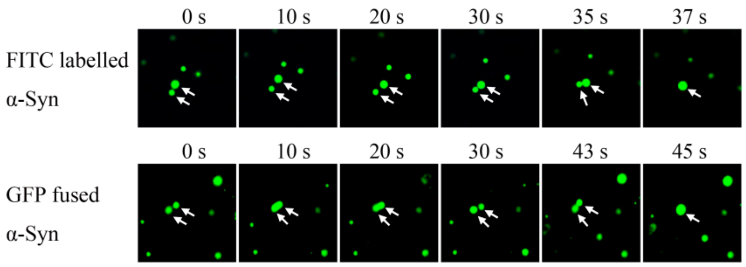

Fig. S3. The droplets fusions of FITC labelled or GFP-fused α-Syn.

The droplets fusions of FITC labelled or GFP-fused α-Syn did not differ significantly. Both fluorescence labelled droplets were highly mobile and underwent frequent fusion events with a rapid relaxation into spherical assemblies.

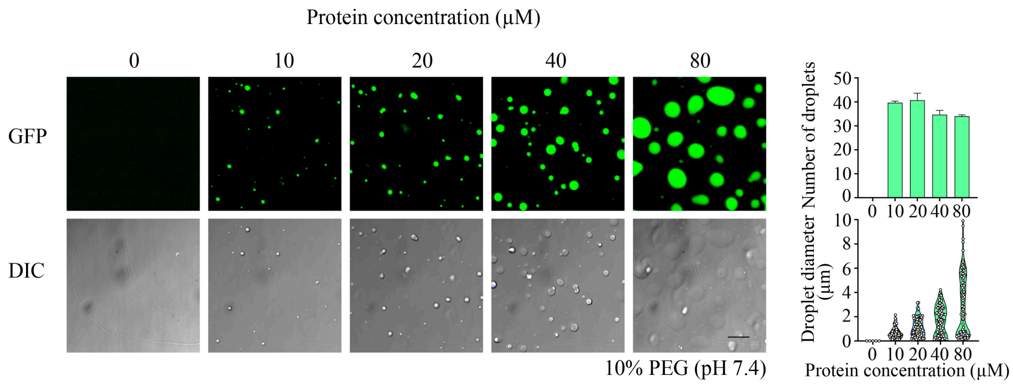

Fig. S4. Effects of protein concentration on the LLPS behavior of α–Syn. Representative fluorescence and differential interference contrast (DIC) images of droplets produced by phase separation of GFP-αSyn incubated at the noted protein concentrations at pH 7.4. (Scale bar, 10 μm).

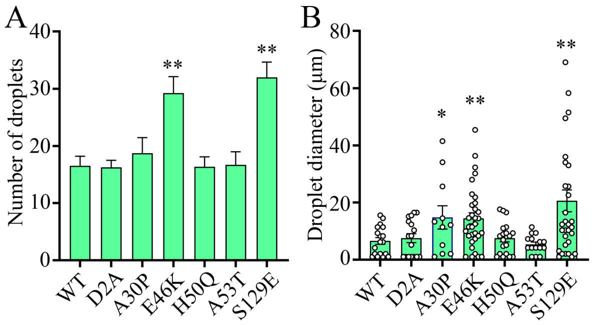

Fig. S5. The mean numbers and diameters of droplets at 7.4 pH in Figure 4 were quantitated using ImageJ.

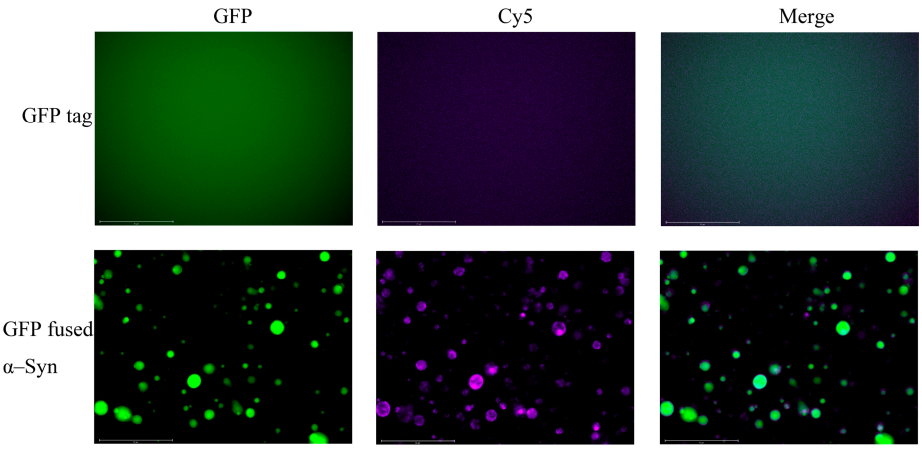

Fig. S6. Co-localization of curcumin and α–Syn during droplets formation. GFP-tagged α–Syn (80 μM) was induced to undergo LLPS in the presence of 10% PEG-6000 and in the presence of 10 μM Cy5.5-curcumin.

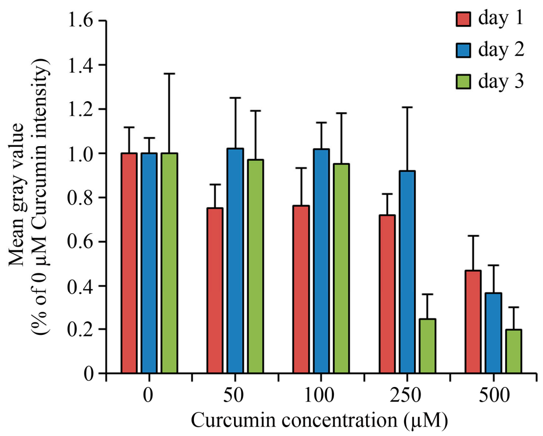

Fig. S7. The dose-dependent effects of curcumin on oligomer formation in Figure 6C were quantitated by scanning for mean grey values (% of 0 μM curcumin intensity) using ImageJ.

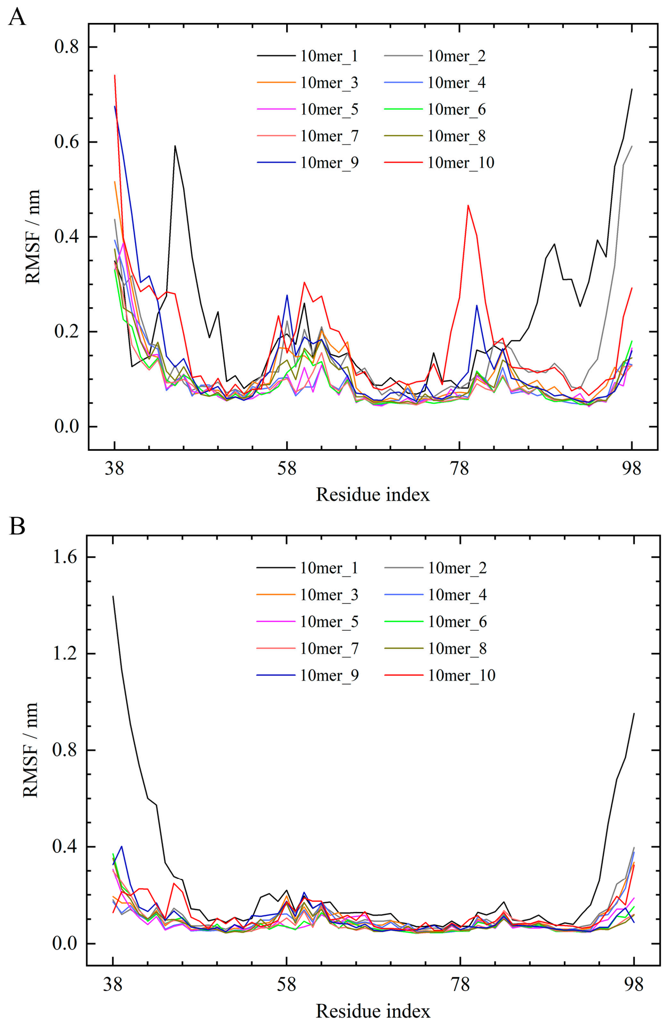

Fig. S8. The root-mean-square fluctuations (RMSFs) of the 10mer α-Syn$^{38\text{-}98}$ with 5 (A) and 10 (B) curcumin molecules bound, respectively.

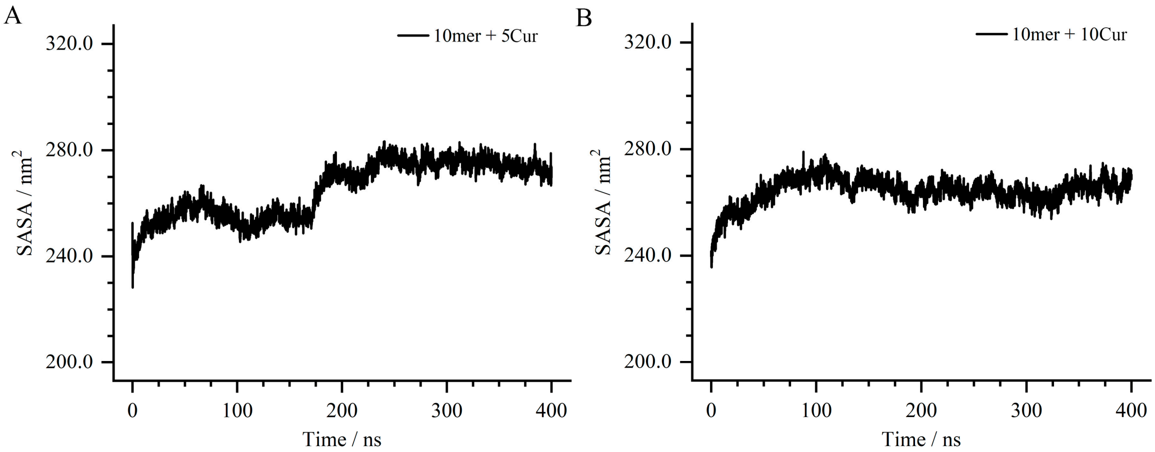

Fig. S9. The solvent accessible surface area (SASA) of the 10mer α-Syn$^{38\text{-}98}$ with 5 (A) and 10 (B) curcumin molecules bound, respectively.

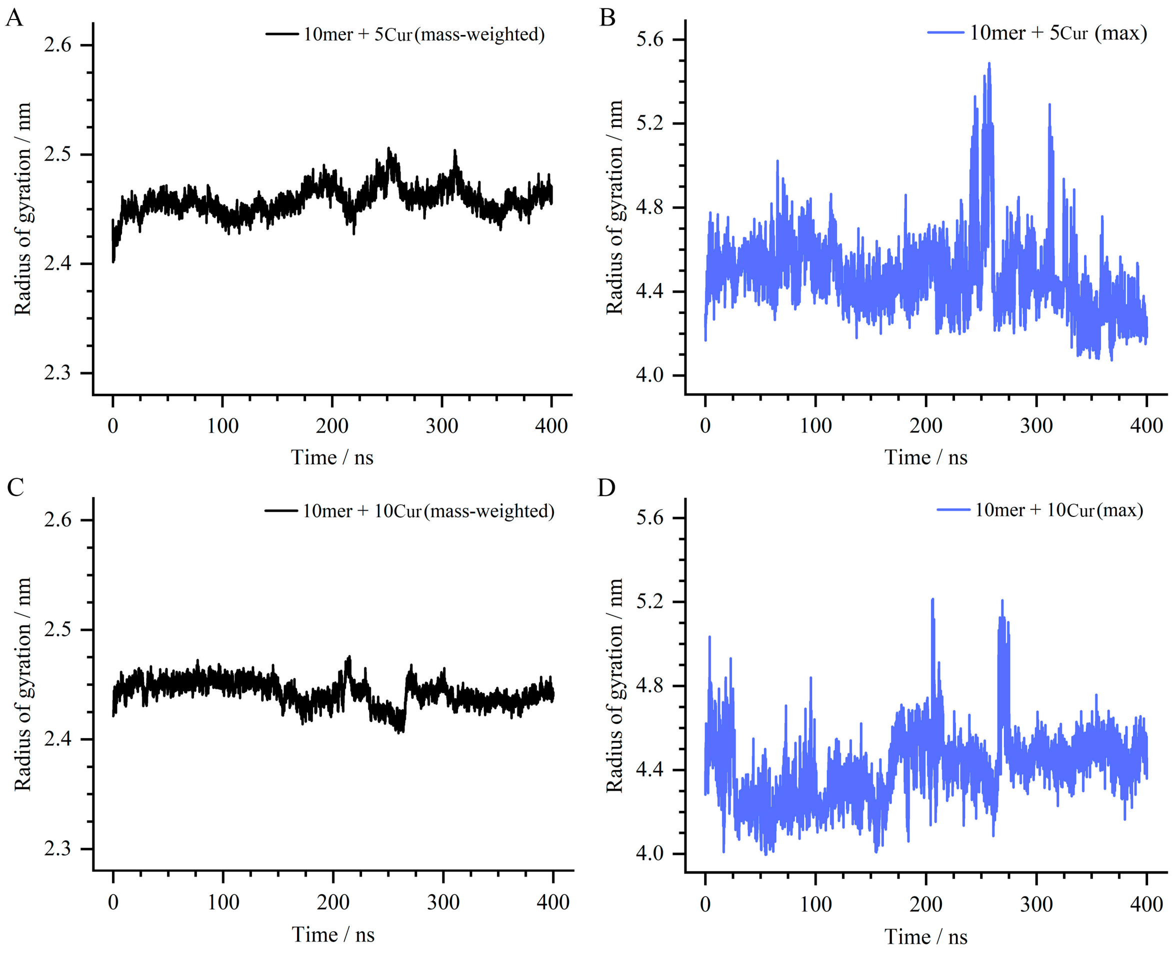

Fig. S10. The mass-weighted and max radius of gyration (Rg) of the 5Cur (A and B) and 10Cur (C and D) bound 10mer α-Syn$^{38-98}$.

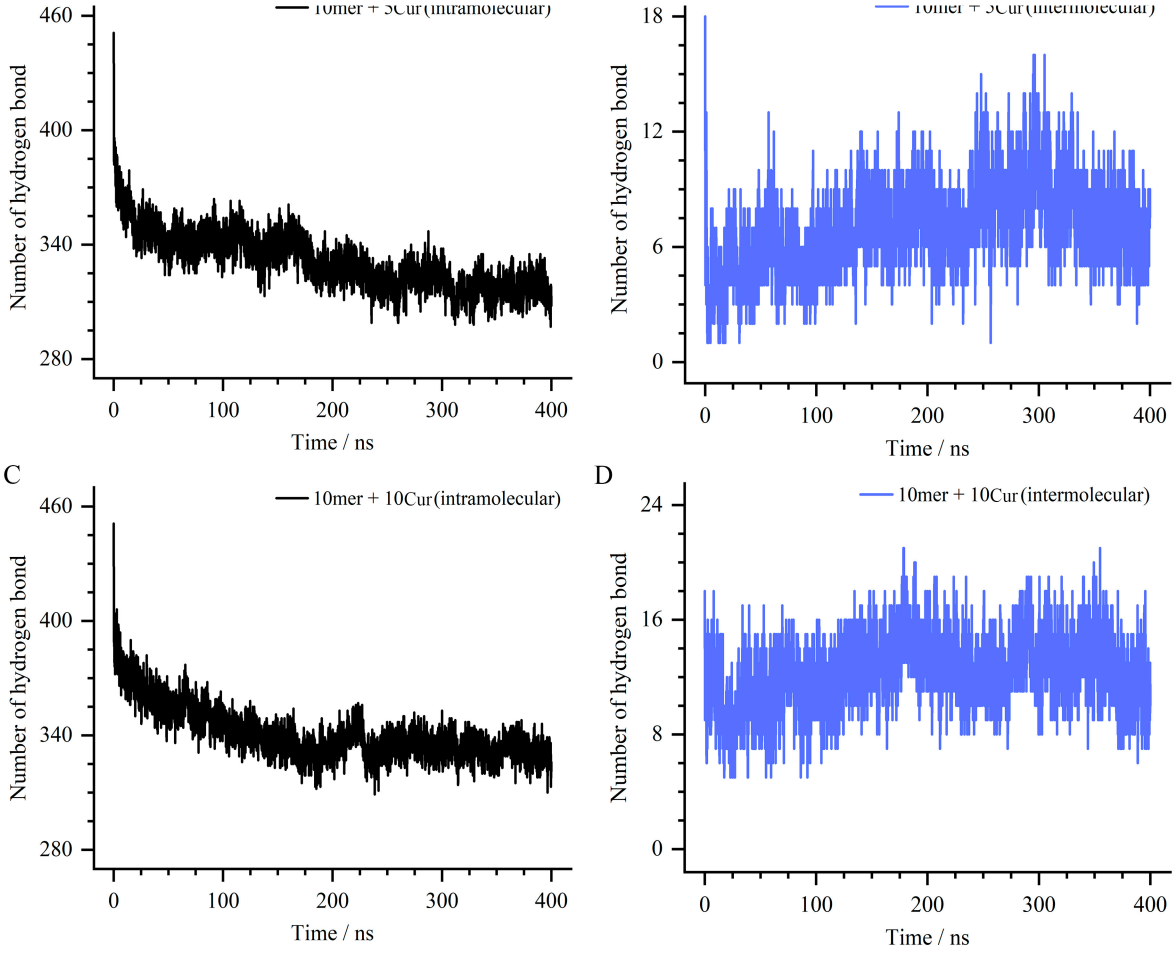

Fig. S11. The intramolecular hydrogen bonds within the backbones of the 5Cur (A) and 10Cur (C) bound 10mer α–Syn$^{38\text{-}98}$ together with the intermolecular hydrogen bonds between 10mer α–Syn$^{38\text{-}98}$ and the binding 5Cur (B) and 10Cur (D).

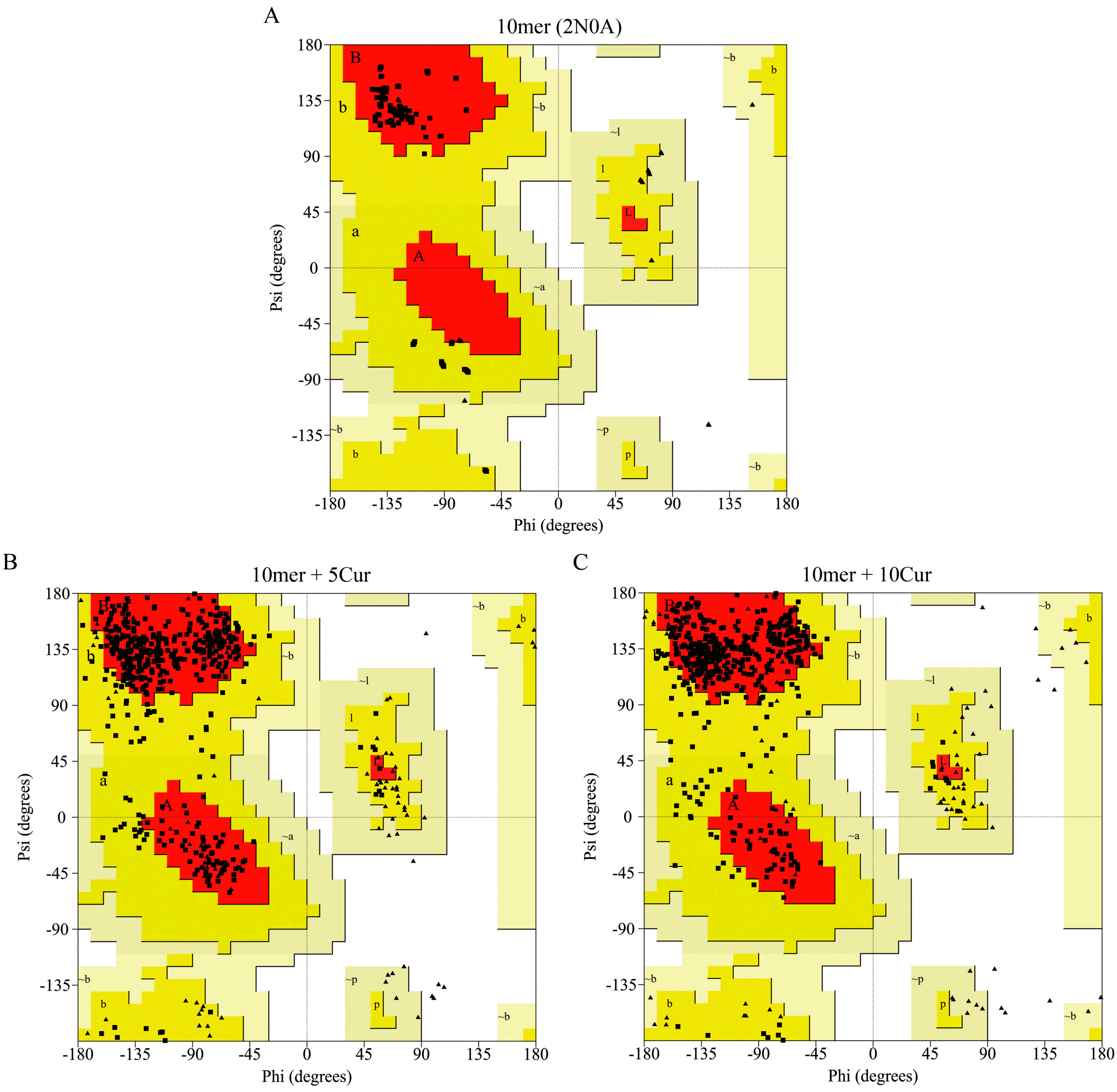

Fig. S12. The Ramachandran plots of the apo and the curcumin bound 10mer α-Syn$^{38\text{-}98}$. The proportions of the residues in most favored regions were 89.4%, 83.8%, and 80.8% for the NMR 10mer structure (2N0A), the 5Cur-bound 10mer, and the 10Cur-bound 10mer, respectively. The figure was generated with the online service of SAVES v6.0 (https://saves.mbi.ucla.edu).
